# Site- and Energy-Selective Low-Energy Electron Emission by X‑rays in the Aqueous Phase

**DOI:** 10.1021/jacs.5c06436

**Published:** 2025-06-13

**Authors:** Dana Bloß, Rémi Dupuy, Florian Trinter, Isaak Unger, Noelle Walsh, Gunnar Öhrwall, Niklas Golchert, Gabriel Klassen, Adrian Krone, Yusaku Terao, Johannes H. Viehmann, Lasse Wülfing, Clemens Richter, Tillmann Buttersack, Lorenz S. Cederbaum, Uwe Hergenhahn, Olle Björneholm, Arno Ehresmann, Andreas Hans

**Affiliations:** † Institut für Physik und CINSaT, 9178 Universität Kassel, Heinrich-Plett-Str. 40, 34132 Kassel, Germany; ‡ Laboratoire de Chimie Physique - Matière et Rayonnement, CNRS, LCP-MR, 27063 Sorbonne Université, 75005 Cedex 05 Paris, France; § Fritz-Haber-Institut der Max-Planck-Gesellschaft, Faradayweg 4-6, 14195 Berlin, Germany; ∥ Department of Physics and Astronomy, Uppsala University, Box 516, 75120 Uppsala, Sweden; ⊥ MAX IV Laboratory, Lund University, Box 118, 22100 Lund, Sweden; # Fakultät Physik, Technische Universität Dortmund, Maria-Goeppert-Mayer-Str. 2, 44227 Dortmund, Germany; ∇ Theoretische Chemie, Institut für Physikalische Chemie, Universität Heidelberg, Im Neuenheimer Feld 229, 69120 Heidelberg, Germany

## Abstract

Low-energy-electron emission from resonant Auger final states via intermolecular Coulombic decay (RA-ICD) has previously been described as a promising scenario for controlling radiation damage for medical purposes, but it has so far only been observed in prototypical atomic and molecular van der Waals dimers and clusters. Here, we report the experimental observation of RA-ICD in an aqueous solution. We show that for solvated Ca^2+^ ions, the emission can be very efficiently controlled by tuning the photon energy of exciting X-rays to inner-shell resonances of the ions. Our results provide the next step from demonstrating RA-ICD in relatively simple prototype systems to understanding the relevance and potential applications of ICD in real-life scenarios.

## Graphical abstract

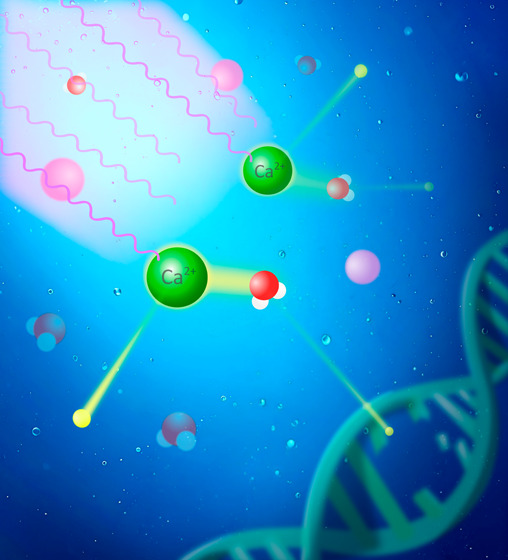

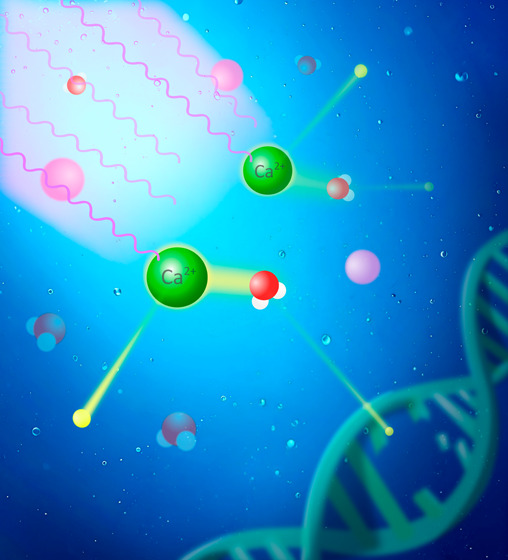

## Introduction

A major challenge in X-ray-based radiation therapies is to achieve the largest possible contrast between the doses of radiation deposited in the malignant versus the surrounding healthy tissue, which the radiation inevitably needs to pass through. About a decade ago, it was suggested that this contrast could be significantly enhanced by making use of a novel mechanism which locally produces radicals and low-energy electrons (LEEs), namely intermolecular Coulombic decay (ICD) of states populated by resonant Auger decay (RA).^1,2^ The reaction products of the RA-ICD process are presumably the main mediators of biological damage by causing single or multiple DNA strand breaks.^3–5^

ICD is a nonlocal autoionization mechanism in which the excess energy of an excited part of an extended system is transferred to a neighbor, thereby ionizing it.^6–9^ First predicted in 1997,^6^ ICD and a number of related processes have by now been observed in a plethora of systems.^9^ While the majority of experimental reports uses photoionization, the initial excitation of the system can proceed via different mechanisms.^9^

The particular variant of interest, RA-ICD, is illustrated in Figure 1 exemplarily for a Ca^2+^ ion with a water molecule as its neighbor, the case which will be studied in the present work. In this scenario, an atom or molecule, which is weakly bound to one or more neighbors, e.g., through van der Waals or hydrogen bonds, is first resonantly inner-shell-excited by X-rays (Figure 1a). The resonant nature of this first step of the overall mechanism ensures a largely higher absorption cross section as compared to absorption due to photoionization into a continuum. A common decay path of such core-excited species is spectator RA decay. Here, a valence electron fills the inner-shell vacancy and another valence electron is emitted, while the initially excited electron stays as a “spectator” in its orbital (Figure 1b). The internal excess energy of the RA final states is typically not enough for further local autoionization. If the atom or molecule does not have neighbors, further decay is only possible radiatively and/or via dissociation. The presence of neighbors, however, enables further autoionization of the system as a whole via ICD: the excited ion decays to its ground state transferring the excess energy to ionize a valence electron from a neighbor (Figure 1c). The emitted electron, the so-called ICD electron, has typically a rather low kinetic energy below 30 eV. The potential relevance of ICD and related phenomena in radiation biology has been discussed intensely.^9–11^

**Figure 1:**
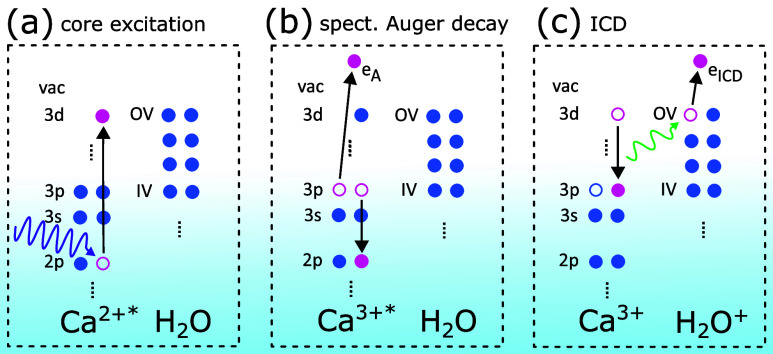
Illustration of the process of interest. (a) Resonant photoexcitation of a Ca^2+^ ion. (b) Spectator resonant Auger decay. (c) Intermolecular Coulombic decay with ionization of a neighboring water molecule. For simplicity, only one water neighbor is shown, but in the experiments the Ca^2+^ ion is fully solvated. Dashed vertical lines indicate energetically lower lying electronic levels, which are not relevant for the present discussion.

Soon after RA-ICD and its potential for medical applications were recognized in pioneering theoretical work^1^ and in an experimental study on molecular van der Waals dimers,^2^ it has also been observed in Ar dimers.^12^ Simultaneously, it was shown that in heterogeneous rare-gas dimers the energies of the emitted LEEs can be adjusted by choosing a neighbor atom with appropriate ionization energy.^13–15^ Despite its intriguing application potential, however, no attempt has been reported to transfer RA-ICD from prototypical van der Waals dimers to more realistic samples. Most studies on liquid samples discuss ICD of electronic inner-shell vacancies, a variant which has been named “core-level ICD”.^16–18^ In this case, ICD directly competes with Auger decay and the ICD electrons have kinetic energies in the range of typical Auger emission (depending on the system under investigation, for Ca ions around 300 eV). It is important to differentiate RA-ICD and core-level ICD as two related mechanisms with yet fundamentally different impact on the photochemistry of the system due to their different reaction products. As demonstrated recently, core-level ICD can, e.g., be applied to study the chemical environment of a solvated ion.^18^ However, it does not directly produce LEEs and is therefore regarded to be less relevant in a biochemical or radiation chemistry context.

From the viewpoint of medical applications, RA-ICD provides an intriguing advantage. Resonant inner-shell excitation with X-rays is highly element-selective, meaning that at a particular resonance energy, photons are nearly exclusively absorbed by one species of atom, while the rest of a system is basically transparent. This can be utilized for targeted energy deposition, e.g., via a marker element, while leaving the surroundings mostly unaffected. The destructive LEEs and radicals are thus produced locally at the point of interest.

A major challenge for the investigation of LEE-emitting ICD and related mechanisms in larger and more complex systems, e.g., in liquids, is the overwhelming background of LEEs due to other processes, mainly electron-impact excitation or ionization and quasi-elastic scattering processes.^19^ In the present study, we overcome this challenge by applying electron–electron coincidence spectroscopy to an aqueous solution and report the first observation of RA-ICD in liquids.

## Results and Discussion

As an explicit example, we consider the 2*p* → 3*d* resonant excitation of solvated Ca^2+^ ions. To obtain information for both the high-resolution resonant Auger spectra as well as the subsequently emitted LEEs, we used the results of two independently performed experiments. These combine the benefits of high-resolution electron spectroscopy using a hemispherical analyzer and low-resolution, but coincident detection using a magnetic-bottle coincidence spectrometer (see Methods section).

In both experiments, the energetic positions of the Ca^2+^ 2*p* → 3*d* resonances were identified by scanning the exciting-photon energy stepwise and recording the total or partial electron yield. Typical electron yield curves are shown in Figure 2. Two prominent resonances attributed to the 2*p*_1/2,3/2_ → 3*d* fine-structure doublet are present.^20^ The weaker side structures, assigned to crystal field effects, have been discussed elsewhere.^20–22^ Note that even after careful photon-energy calibration, a slight deviation on the order of 200 meV in resonance energies remains between the two experiments, with neither exactly matching previously reported values.^20^ The origin of this deviation cannot be reconstructed. It is, however, most likely a result of the fact that the calibrations were done not exactly in the energy range of the Ca L-edge and can be regarded as uncertainty for the observed resonance energies. Since the resonances were identified in each experiment individually, this does not affect our conclusions in any way.

**Figure 2:**
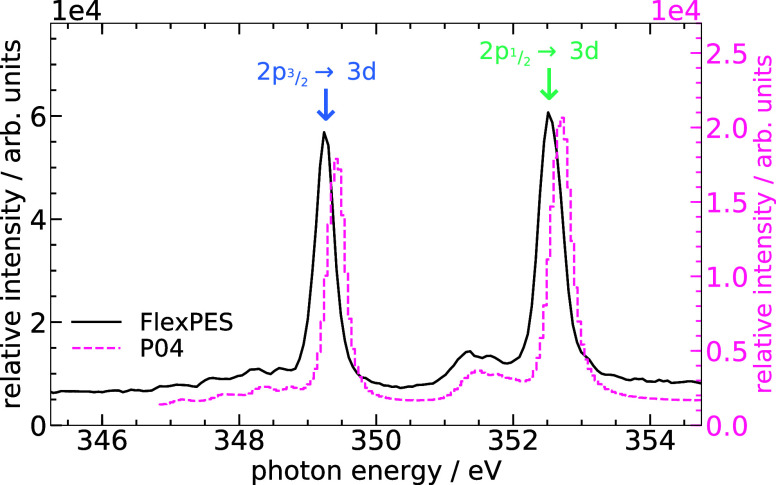
Total electron yield (FlexPES, black solid line and left *y* axis) and partial electron yield (P04, magenta dashed line, right *y* axis, based on data from ref 18, reused under CC BY 4.0.) as a function of the exciting-photon energy across the Ca^2+^ 2*p* edge from both conducted experiments. A slight shift is observed between the experiments at P04 and FlexPES, respectively, which is most likely a result of energy-calibration uncertainties (see text).

Using high-resolution electron spectroscopy with the hemispherical analyzer, the resonant Auger spectra on both resonances and well above the 2*p* edge (binding energies of 352.8 and 356.6 eV for the 2*p*_3/2_ and 2*p*_1/2_ components^16^) were measured and are displayed in Figure 3. The nonresonant Auger spectrum, shown in Figure 3b, has been reported and discussed earlier^16,17^ and serves as a reference for the interpretation of the spectra recorded on the resonances. It contains a main contribution of conventional Auger electrons between 280 and 290 eV and a weaker signal between 300 and 310 eV attributed to core-level ICD. This latter variant of ICD is a direct competitor to Auger decay, a valence electron from Ca fills the core vacancy and a valence electron from a neighboring water molecule is emitted.^16^ Relative to the nonresonant Auger spectrum, the features in the two spectra on the resonances are rather straightforward to assign. The fastest electrons in the range from 330 eV to about 340 eV are mainly valence electrons from water. The sharp prominent peaks at 318.7 eV (2*p*_3/2_ resonance) and 322.1 eV (2*p*_1/2_ resonance) represent the 3*p* photoelectrons from Ca^2+^ with a binding energy of 29.8 eV.^16^ Their intensity is resonantly enhanced due to participator resonant Auger decay. At about 19 eV lower kinetic energy, another relatively sharp peak can be attributed to the Ca 3*s* photoelectrons. In between the 3*s* and 3*p* photoelectrons, there is a broader peak which originates from resonant core-level ICD, which has recently been analyzed in detail.^18^ In the region between about 282 and 295 eV, the spectator resonant Auger spectrum corresponding to transitions from Ca^2+^(2*p* ^–1^3*d*) to Ca^3+^(3*p* ^–2^3*d*) configurations can be observed, with a characteristic “spectator shift”^17^ compared to the nonresonant Auger spectrum. At even lower kinetic energies transitions to final states with holes in the 3*s* level occur.

**Figure 3:**
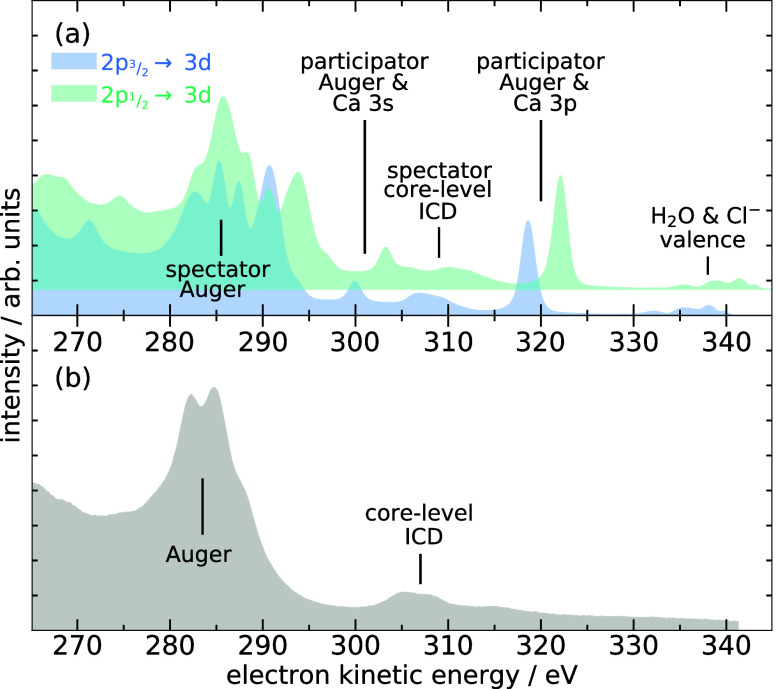
(a) Resonant Auger spectra after Ca^2+^ 2*p*_3/2_ → 3*d* (349.4 eV, blue) and 2*p*_1/2_ → 3*d* (352.7 eV, green) photoexcitation. An offset has been applied to the latter for better visibility. (b) Auger spectrum after 2*p* photoionization, recorded at 460 eV exciting-photon energy. The main features are labeled, for detailed discussion see text. Based on data from ref 18, reused under CC BY 4.0.

The remaining internal energy in the Ca^3+^ ion can easily be estimated from the difference between the spectator resonant Auger region and the 3*p* photoelectron peak in the kinetic-energy spectrum, which represents the ionic ground state. This energy amounts to about 28 to 37 eV and is available to be transferred to neighbors. The kinetic energies of electrons emitted in ICD of these states can then be coarsely calculated. The ionization energy of water (11.3 eV in liquid phase^23^) and the potential Coulomb energy between the resulting ions need to be subtracted from the available excess energy. The Coulomb energy between the Ca^3+^ and the H_2_O^+^ ion strongly depends on their interionic distance and the screening by the environment. From the average Ca–O distance of 2.46 Å^24^ a nominal Coulomb energy of 17.6 eV can be calculated,^16^ while at infinite distance or for complete screening it is zero. Experimentally, the Coulomb energy can be deduced from the energetic distance between Auger and core-level ICD features in previous studies or from the present kinetic energies in the spectra in Figure 3. It turns out that there is almost perfect screening by the environment and that the two-site ionization potentials of the ICD final states are close to the sum of the ionization energies of the individual ions.^16^ Consequently, a Coulomb energy of at most a few eV needs to be considered. Note that the final ionic states of core-level ICD and RA-ICD are identical, namely Ca^3+^ and H_2_O^+^. Under these considerations we expect an RA-ICD spectrum centered at about 20 eV and of about 10 eV width. Except for slight changes in the spectral structure the energy range is expected to be independent from the fine-structure component, since this difference is mainly taken by the spectator Auger electron.

This estimated kinetic-energy range of RA-ICD electrons lies energetically within the typical background of LEEs observed in electron spectra from liquids.^19^ Without further discrimination, the identification of a broad feature in this range is extremely challenging using conventional electron spectroscopy. We therefore applied electron–electron coincidence spectroscopy to reduce the background of inelastically scattered electrons tremendously. Due to the large photoexcitation cross section on the Ca^2+^ 2*p* resonances, the resonant Auger electrons in the range between 282 and 295 eV can be unambiguously identified in the electron spectrum,^25^ although the resolution is significantly inferior to the hemispherical analyzer. We then select events of two-electron coincidences (resonant Auger electron and RA-ICD electron). In Figure 4, the kinetic-energy spectra of the second, slower electron are plotted for both resonances with the coincidence condition that the first electron was the spectator Auger electron. The off-resonant spectrum of single-electron events serves as a reference. The spectra have been normalized to their maximum to allow for comparison of spectral features within a single graph and additionally are plotted in a magnified presentation to emphasize features in the region of lower intensity above 10 eV. The expected energy range (15–25 eV) of the RA-ICD electrons has been highlighted. For a more detailed description of the coincidence data treatment see the Supporting Information.^25^

**Figure 4:**
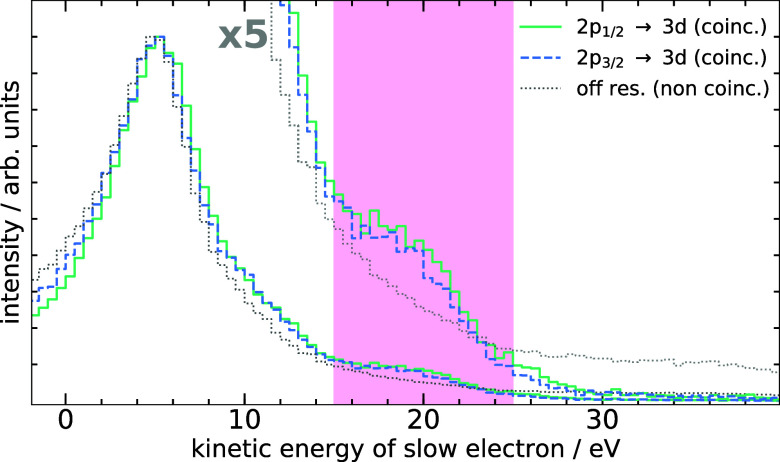
LEE spectra of a 4 M CaCl_2_ solution exposed to X-rays of different energies. The gray dotted curve is a reference spectrum of single-electron events above the Ca^2+^ 2*p* ionization threshold taken at 405 eV exciting-photon energy. The blue dashed line and the green solid line represent spectra on the 2*p*_3/2_ → 3*d* and 2*p*_1/2_ → 3*d* resonances at 349.25 and 352.5 eV, respectively. The spectra on the resonances were filtered using the coincidence condition that the first of two coincidentally detected electrons is the Ca^2+^ 2*p* → 3*d* spectator resonant Auger electron. All spectra are normalized to their maximum and are additionally plotted magnified to emphasize the low-intensity region. The red shaded area indicates the expected range of the RA-ICD electrons.

It is evident that also the coincidence filtering cannot completely suppress the LEE tail increasing toward zero kinetic energy, which is omnipresent in the spectrum of the second electron. Note that the spectral shape close to zero eV may not be accurate due to the difficulty of measuring very slow electrons from a liquid.^19^ Importantly, however, on top of the LEE background, for both resonances a significant structure can be observed which perfectly matches the estimated RA-ICD kinetic-energy range and is absent in the reference spectrum. We thus conclude that the observed feature between about 15 and 25 eV kinetic energy originates from RA-ICD of solvated Ca^3+^ ions. A second feature may be identified around 10 eV, which gradually merges into the LEE background and is therefore less discernible. Its origin cannot be unambiguously identified from the present data and could possibly include RA-ICD ionizing water valence orbitals with higher binding energies, or electron-transfer-mediated decay of Ca^3+^ ions subsequent to RA-ICD.^26,27^

It is worth mentioning that for concentrations of about 4 M, as used in the present coincidence experiment, the effect of ion pairing has been observed.^18^ That means, that the counterion (Cl^–^) is present in the first solvation shell and can be ionized through any ICD variant as well as water molecules. The contribution attributed to ion pairing is, however, weak^18^ and since the coincidence spectra were only taken at one concentration, it cannot be identified here. We suggest that future studies should address the impact of ion pairing on RA-ICD.

While the feature assigned to RA-ICD may seem rather weak compared to the remaining LEE background, it is important, however, to consider this in proper context. First, it is remarkable that the RA-ICD feature contrasts so clearly from the reference spectrum at all. All other works reporting LEE emission from liquids after soft X-ray irradiation used some background-subtraction method to make the features of interest visible (e.g., refs. 26,27 ). Second, the experiment integrates over an interaction and acceptance volume (unfortunately not sharply delimited), in which much more water molecules than Ca ions are present. In the immediate environment of the ions, the emission of RA-ICD electrons should be more efficient than the average LEE emission. A quantification of this is not possible here and should be subject to future investigations.

Regarding the efficiency of the RA-ICD process itself, for van-der-Waals-bound dimers, RA-ICD was found to be very efficient, occurring on a time scale below 20 fs.^2^ A similar time range was reported for direct core-level ICD of 2p-ionized solvated Ca ions.^16^ ICD of valence-ionized states in Mg ions was predicted to be even faster, below 1 fs.^28^ Although none of these cases is equivalent to the presently discussed variant of ICD and a quantification is not possible from the present data, it is reasonable to assume a similar lifetime here.

We emphasize the resonant and site-selective character of the LEE emission triggered by RA-ICD, which was the fundamental idea in refs. 1,2 to suggest its application for medical purposes. Through the resonant excitation as a precondition for RA-ICD, the production of LEEs and radicals should only appear in a very narrow window of the X-ray photon energy (sub 1 eV wide in the present case). Naturally, the LEEs originating from RA-ICD are only emitted locally from the immediate surroundings of the Ca ions. While Ca is abundant in the biosphere, the scenario could be transferred to another marker element, which may artificially be introduced into an organism, enriched, e.g., in cancer tissue. Choosing the resonant photon energy of the marker element, destructive LEEs can then be produced efficiently and locally, while the rest of the tissue is basically transparent for the X-rays. Higher atomic number *Z* will increase the contrast/selectivity, as the photoionization cross section for C, N, and O drops with increasing photon energy. Moreover, deep core-level vacancies in high-*Z* elements give rise to more electrons due to cascade decay.^26^ It should also be noted that the damage caused by LEEs depends on their kinetic-energy distribution. For example, the impact of electrons with different kinetic energies may mainly cause single, double, or multiple strand breaks to DNA.^3–5,29^ In general, the distribution of kinetic energies of electrons emitted due to RA-ICD can be steered by choosing an appropriate system.

## Methods

High-resolution Auger spectra were recorded using a hemispherical electron analyzer coupled to a liquid microjet device.^30,31^ The instrument was installed at the P04 beamline^32^ of the PETRA III synchrotron facility (DESY, Hamburg, Germany). A 1.5 M solution of CaCl_2_ was prepared by dissolving a commercial salt (Sigma-Aldrich, 99% purity) in ultrapure water (resistivity 18.2 MΩ/cm) and subsequent filtration and degassing. For typical operation conditions of the liquid microjet we used 30 μm diameter glass nozzles and a flow rate of 0.8 mL/min. All spectra were recorded by applying a −50 V bias voltage to the liquid jet, allowing to suppress water gas-phase contributions to the spectra.^23^ The photon energy of the beamline was calibrated against known gas-phase K-edge absorption lines of different gases (CO, N_2_, SF_6_, and Ne). We operated with a photon bandwidth of about 60 meV and an analyzer resolution of 200 meV, which is sufficient to resolve all features in the Auger spectra.

For monitoring LEE emission occurring subsequently to resonant Auger decay we used a setup for electron–electron coincidence spectroscopy.^26,33^ This experiment was performed at the FlexPES beamline at the MAX IV Laboratory in Lund, Sweden^34^ during single-bunch delivery. A magnetic-bottle time-of-flight electron spectrometer was used to efficiently record coincidences of two electrons emitted in pairs after a single excitation. Details of the setup are described elsewhere.^26,33^ A 4 M solution of CaCl_2_ was prepared by the same procedure as in the first experiment. The sample was introduced into vacuum at a temperature of 4 °C and at flow rates between 0.6 and 0.8 mL/min. For measuring the LEE spectra, a +26 V bias voltage was applied to the drift tube of the magnetic bottle in order to accelerate electrons with near zero kinetic energy such that they arrive at the detector within the time window between two consecutive pulses (320 ns) and a −3 V bias voltage was applied to the jet itself. The exciting-photon energy axis was calibrated at the Ar L-edge and the time-of-flight axis was converted to kinetic energies by measuring water O 1s photoemission (for which the binding energy is known) at various exciting-photon energies. Using a procedure of data acquisition during several consecutive exciting-photon pulses, the relevance of random coincidences was estimated to be negligible.^25^

## Supplementary Material



## Data Availability

The data generated in this study have been deposited in a Zenodo database [10.5281/zenodo.13358738].

## Notes

Time-of-flight and kinetic-energy electron coincidence spectra of CaCl_2_ (PDF)

## Notes

For the data evaluation freely available, common python packages were used. The developed code is available from the corresponding authors upon request.

## References

[1] Gokhberg K., Kolorenč P., Kuleff A. I., Cederbaum L. S. (2014). Site- and
energy-selective slow-electron production through intermolecular Coulombic
decay. Nature.

[2] Trinter F., Schöffler M. S., Kim H.-K., Sturm F. P., Cole K., Neumann N., Vredenborg A., Williams J., Bocharova I., Guillemin R., Simon M., Belkacem A., Landers A. L., Weber T., Schmidt-Böcking H., Dörner R., Jahnke T. (2014). Resonant Auger decay driving intermolecular Coulombic
decay in molecular dimers. Nature.

[3] Boudaïffa B., Cloutier P., Hunting D., Huels M. A., Sanche L. (2000). Resonant Formation
of DNA Strand Breaks by Low-Energy (3 to 20 eV) Electrons. Science.

[4] Alizadeh E., Sanche L. (2012). Precursors of Solvated Electrons
in Radiobiological
Physics and Chemistry. Chem. Rev..

[5] Alizadeh E., Orlando T. M., Sanche L. (2015). Biomolecular
Damage Induced by Ionizing
Radiation: The Direct and Indirect Effects of Low-Energy Electrons
on DNA. Annu. Rev. Phys. Chem..

[6] Cederbaum L. S., Zobeley J., Tarantelli F. (1997). Giant Intermolecular
Decay and Fragmentation
of Clusters. Phys. Rev. Lett..

[7] Marburger S., Kugeler O., Hergenhahn U., Möller T. (2003). Experimental
Evidence for Interatomic Coulombic Decay in Ne Clusters. Phys. Rev. Lett..

[8] Jahnke T., Czasch A., Schöffler M. S., Schössler S., Knapp A., Käsz M., Titze J., Wimmer C., Kreidi K., Grisenti R. E., Staudte A., Jagutzki O., Hergenhahn U., Schmidt-Böcking H., Dörner R. (2004). Experimental
Observation of Interatomic Coulombic Decay in Neon Dimers. Phys. Rev. Lett..

[9] Jahnke T., Hergenhahn U., Winter B., Dörner R., Frühling U., Demekhin P. V., Gokhberg K., Cederbaum L. S., Ehresmann A., Knie A., Dreuw A. (2020). Interatomic and Intermolecular
Coulombic Decay. Chem. Rev..

[10] Mucke M., Braune M., Barth S., Förstel M., Lischke T., Ulrich V., Arion T., Becker U., Bradshaw A., Hergenhahn U. (2010). A hitherto
unrecognized source of
low-energy electrons in water. Nat. Phys..

[11] Hergenhahn U. (2012). Production
of low kinetic energy electrons and energetic ion pairs by Intermolecular
Coulombic Decay. Int. J. Radiat. Biol..

[12] Kimura M., Fukuzawa H., Sakai K., Mondal S., Kukk E., Kono Y., Nagaoka S., Tamenori Y., Saito N., Ueda K. (2013). Efficient site-specific
low-energy electron production via interatomic
Coulombic decay following resonant Auger decay in argon dimers. Phys. Rev. A.

[13] Kimura M., Fukuzawa H., Tachibana T., Ito Y., Mondal S., Okunishi M., Schöffler M., Williams J., Jiang Y., Tamenori Y., Saito N., Ueda K. (2013). Controlling Low-Energy
Electron Emission via Resonant-Auger-Induced Interatomic Coulombic
Decay. J. Phys. Chem. Lett..

[14] Miteva T., Chiang Y.-C., Kolorenč P., Kuleff A. I., Cederbaum L. S., Gokhberg K. (2014). The effect of the partner
atom on the spectra of interatomic
Coulombic decay triggered by resonant Auger processes. J. Chem. Phys..

[15] O’Keeffe P., Ripani E., Bolognesi P., Coreno M., Devetta M., Callegari C., Di Fraia M., Prince K. C., Richter R., Alagia M., Kivimäki A., Avaldi L. (2013). The Role of the Partner
Atom and Resonant Excitation Energy in Interatomic Coulombic Decay
in Rare Gas Dimers. J. Phys. Chem. Lett..

[16] Pokapanich W., Kryzhevoi N. V., Ottosson N., Svensson S., Cederbaum L. S., Öhrwall G., Björneholm O. (2011). Ionic-Charge Dependence of the Intermolecular
Coulombic Decay Time Scale for Aqueous Ions Probed by the Core-Hole
Clock. J. Am. Chem. Soc..

[17] Ottosson N., Öhrwall G., Björneholm O. (2012). Ultrafast charge delocalization dynamics
in aqueous electrolytes: New insights from Auger electron spectroscopy. Chem. Phys. Lett..

[18] Dupuy R., Buttersack T., Trinter F., Richter C., Gholami S., Björneholm O., Hergenhahn U., Winter B., Bluhm H. (2024). The solvation
shell probed by resonant intermolecular Coulombic decay. Nat. Commun..

[19] Malerz S., Trinter F., Hergenhahn U., Ghrist A., Ali H., Nicolas C., Saak C.-M., Richter C., Hartweg S., Nahon L., Lee C., Goy C., Neumark D. M., Meijer G., Wilkinson I., Winter Thürmer., Thürmer S. (2021). Low-energy constraints on photoelectron
spectra measured
from liquid water and aqueous solutions. Phys.
Chem. Chem. Phys..

[20] Abid A. R., Mailhiot M., Boudjemia N., Pelimanni E., Milosavljević A. R., Saak C.-M., Huttula M., Björneholm O., Patanen M. (2021). The effect of relative humidity on
CaCl_2_ nanoparticles studied by soft X-ray absorption spectroscopy. RSC Adv..

[21] Yang F., Liu Y.-S., Feng X., Qian K., Kao L. C., Ha Y., Hahn N. T., Seguin T. J., Tsige M., Yang W., Zavadil K. R., Persson K. A., Guo J. (2020). Probing calcium solvation
by XAS, MD and DFT calculations. RSC Adv..

[22] Rubensson J.-E., Eisebitt S., Nicodemus M., Böske T., Eberhardt W. (1994). Electron correlation in CaF_2_ studied in
threshold-excited soft-x-ray fluorescence. Phys.
Rev. B.

[23] Winter B., Thürmer S., Wilkinson I. (2023). Absolute Electronic Energetics and
Quantitative Work Functions of Liquids from Photoelectron Spectroscopy. Acc. Chem. Res..

[24] Jalilehvand F., Spångberg D., Lindqvist-Reis P., Hermansson K., Persson I., Sandström M. (2001). Hydration
of the Calcium Ion. An
EXAFS, Large-Angle X-ray Scattering, and Molecular Dynamics Simulation
Study. J. Am. Chem. Soc..

[25] Bloß, D. Supplemental Material: Site- and Energy-Selective Low-Energy Electron Emission by X-rays in the Aqueous Phase. J. Am. Chem. Soc. 2025. 10.48550/arXiv.2408.12435 PMC12203587 40513115

[26] Bloß D., Trinter F., Unger I., Zindel C., Honisch C., Viehmann J., Kiefer N., Marder L., Küstner-Wetekam C., Heikura E., Cederbaum L. S., Björneholm O., Hergenhahn U., Ehresmann A., Hans A. (2024). X-ray radiation damage
cycle of solvated inorganic ions. Nat. Commun..

[27] Gopakumar G., Unger I., Slavíček P., Hergenhahn U., Öhrwall G., Malerz S., Céolin D., Trinter F., Winter B., Wilkinson I., Caleman C., Muchová E., Björneholm O. (2023). Radiation
damage by extensive local water ionization from two-step electron-transfer-mediated
decay of solvated ions. Nat. Chem..

[28] Stumpf V., Gokhberg K., Cederbaum L. S. (2016). The role
of metal ions in X-ray-induced
photochemistry. Nat. Chem..

[29] Orlando T. M., Oh D., Chen Y., Aleksandrov A. B. (2008). Low-energy electron diffraction and
induced damage in hydrated DNA. J. Chem. Phys..

[30] Winter B., Faubel M. (2006). Photoemission from Liquid Aqueous Solutions. Chem. Rev..

[31] Malerz S., Haak H., Trinter F., Stephansen A. B., Kolbeck C., Pohl M., Hergenhahn U., Meijer G., Winter B. (2022). A setup for studies of photoelectron
circular dichroism from chiral molecules in aqueous solution. Rev. Sci. Instrum..

[32] Viefhaus J., Scholz F., Deinert S., Glaser L., Ilchen M., Seltmann J., Walter P., Siewert F. (2013). The Variable Polarization
XUV Beamline P04 at PETRA III: Optics, mechanics and their performance. Nucl. Instrum. Methods Phys. Res. A.

[33] Pohl M. N., Richter C., Lugovoy E., Seidel R., Slavíček P., Aziz E. F., Abel B., Winter B., Hergenhahn U. (2017). Sensitivity
of Electron Transfer Mediated Decay to Ion Pairing. J. Phys. Chem. B.

[34] Preobrajenski A., Generalov A., Öhrwall G., Tchaplyguine M., Tarawneh H., Appelfeller S., Frampton E., Walsh N. (2023). FlexPES: a
versatile soft X-ray beamline at MAX IV Laboratory. J. Synchrotron Radiat..

