# Supplementary material for: Site- and Energy-Selective Low-Energy Electron Emission by X‑rays in the Aqueous Phase
Source: J Am Chem Soc. 2025 Jun 13;147(25):22115–20. doi: 10.1021/jacs.5c06436 (PMC12203587; doi:10.1021/jacs.5c06436)
Supplement: Supplementary file 1 [file ja5c06436_si_001.pdf]

Supplementary Information for manuscript:
Site- and Energy-Selective Low-Energy Electron Emission by X-rays in the Aqueous Phase

Dana Bloß*,[1] Rémi Dupuy,[2] Florian Trinter,[3] Isaak Unger,[4] Noelle Walsh,[5]
Gunnar Öhrwall,[5] Niklas Golchert,[1] Gabriel Klassen,[1] Adrian Krone,[1] Yusaku Terao,[1]
Johannes H. Viehmann,[1] Lasse Wülfing,[6] Clemens Richter,[3] Tillmann Buttersack,[3] Lorenz S. Cederbaum,[7]
Uwe Hergenhahn,[3] Olle Björneholm,[4] Arno Ehresmann,[1] and Andreas Hans+[1]

[1] *Institut für Physik und CINSaT, Universität Kassel, Heinrich-Plett-Str. 40, 34132 Kassel, Germany*

[2] *Laboratoire de Chimie Physique - Matière et Rayonnement, Sorbonne Université, CNRS, LCP-MR, 75005 Paris Cedex 05, France*

[3] *Fritz-Haber-Institut der Max-Planck-Gesellschaft, Faradayweg 4-6, 14195 Berlin, Germany*

[4] *Department of Physics and Astronomy, Uppsala University, Box 516, 75120 Uppsala, Sweden*

[5] *MAX IV Laboratory, Lund University, Box 118, 22100 Lund, Sweden*

[6] *Fakultät Physik, Technische Universität Dortmund, Maria-Goeppert-Mayer-Str. 2, 44227 Dortmund, Germany*

[7] *Theoretische Chemie, Institut für Physikalische Chemie, Universität Heidelberg, Im Neuenheimer Feld 229, 69120 Heidelberg, Germany*

* dana.bloss@uni-kassel.de

+ hans@physik.uni-kassel.de

ESTIMATE OF THE RELEVANCE OF RANDOM COINCIDENCES

If more than one interaction event occurs from a single exciting-photon pulse, this can cause random coincidences. This can be avoided by keeping the event rate f_{event} much lower than the repetition rate f_{exc} of the excitation, i.e., $f_{event} \ll f_{exc}$. For double-electron coincidences the occurrence of random coincidences can be monitored by setting the acquisition time of the time-to-digital converter (TDC) longer than at least double the temporal spacing between two consecutive exciting-photon pulses. If then a filter condition is applied to the first of two detected electrons, random coincidences will appear as a periodic pattern in the time-of-flight spectrum of the second detected electron with the same periodicity as the excitation pulses (320 ns in the present case). During the experiment at the FlexPES beamline, the acquisition time was set to 1600 ns, i.e., covering 5 pulses. In Fig. S1, we show the time-of-flight spectrum of the second electron detected in two-electron coincidence events without any further filter and with the condition that the first detected electron is the Ca^{2+} resonant Auger electron (for the $2p_{1/2} \rightarrow 3d$ resonance). As expected, without any filter the spectrum shows periodic structures repeating every 320 ns. Under the filter condition, all but one feature disappear. The remaining feature originates from true and random coincidences with the electrons within the filter window. Random coincidences would appear as a periodic structure, which is practically absent. From this we conclude that random coincidences can be neglected in the data treatment.

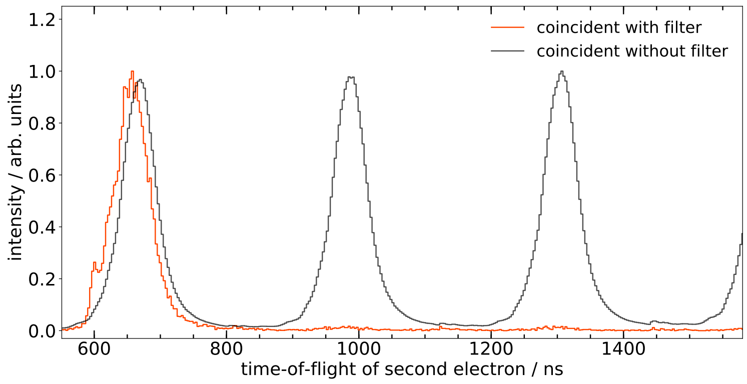

FIG. S1. Time-of-flight spectra of the second detected electron in two-electron-coincidence events on the $2p_{1/2} \rightarrow 3d$ resonance. The black trace shows all second electrons as measured, yielding an expected periodicity of 320 ns. For the red trace, a filter condition was applied for the first electron detected being the resonant Auger electron. The periodicity disappears, indicating that random coincidences are negligible.

IDENTIFICATION OF THE RESONANT AUGER ELECTRONS IN THE TIME-OF-FLIGHT SPECTRA

In the coincidence experiment using the magnetic-bottle time-of-flight spectrometer, the absolute energy resolution decreases for faster electrons, i.e., for electrons of higher kinetic energy. It is therefore essential to identify the spectator resonant Auger electrons unambiguously, which are subsequently used as a filtering condition in the coincidence data set. For that purpose, we measured electron spectra on the Ca^{2+} $2p \rightarrow 3d$ resonances and above the ionization threshold and compared them to the high-resolution spectra obtained with the hemispherical analyzer. An exemplary spectrum is shown in Fig. S2. A clear peak can be identified at 280-290 eV kinetic energy in the spectrum on resonance, which is less pronounced and slightly shifted to lower energies in the spectrum above threshold. Comparison to the spectra in Fig. 3 in the main manuscript clearly allows the assignment of this feature to spectator resonant Auger electrons. The spectra in Fig. S2 comprise events in which only one electron has been detected.

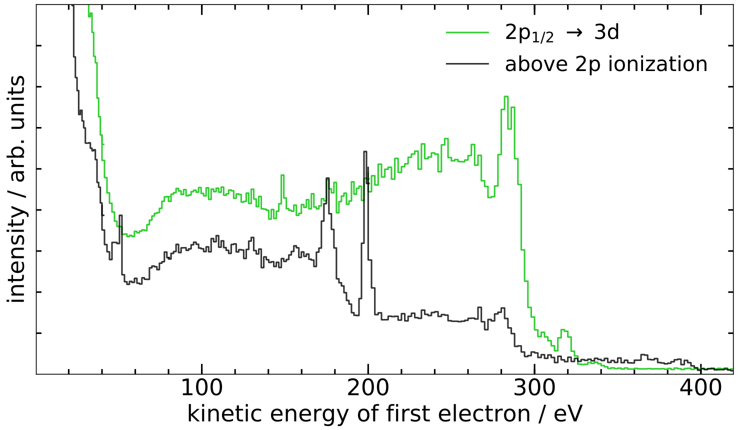

FIG. S2. Electron spectra of a 4 M $CaCl_2$ solution recorded on the $2p_{1/2} \rightarrow 3d$ resonance (green trace) and above the $2p$ ionization threshold (405 eV, black trace). In the spectrum on resonance, the spectator resonant Auger electrons can readily be identified at 280-290 eV kinetic energy. In addition, the participator Auger emission can be observed at about 320 eV, see also Fig. 3 of the main manuscript. Features in the 150-200 eV kinetic-energy range can be assigned to Cl^- $2p$ photoelectrons and Auger electrons, and the peak at about 50 eV in the above-threshold spectrum to Ca^{2+} $2p$ photoelectrons. The intensity of the spectra has been scaled for better presentability, a quantitative comparison is not meaningful due to the different absorption cross sections.

The two-dimensional electron-electron coincidence maps recorded on the $2p_{3/2} \rightarrow 3d$ and $2p_{1/2} \rightarrow 3d$ resonances are displayed in Fig. S3. Such maps are histograms showing all double-electron coincidence events plotted with the kinetic energy of the first electron on the x axis and the kinetic energy of the second electron on the y axis. The low-energy electron (LEE) tail characteristic for all experiments on liquids is very prominent. All electrons in coincidence with a spectator resonant Auger electron appear in a column at 280-290 eV on the x axis. The feature assigned to ICD following resonant Auger decay (RA-ICD) is indicated by arrows in both maps.

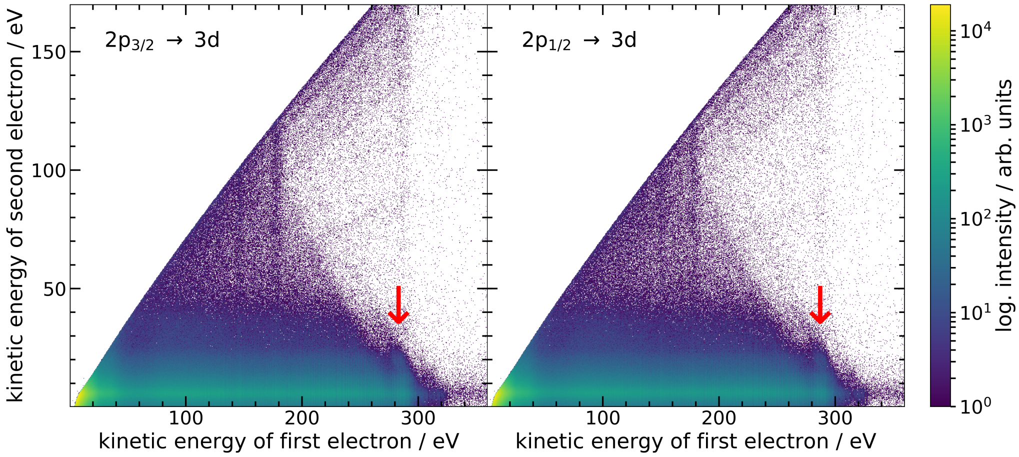

FIG. S3. Electron-electron coincidence maps recorded on the $2p_{3/2} \rightarrow 3d$ (left) and $2p_{1/2} \rightarrow 3d$ (right) resonances. The red arrows indicate the feature attributed to RA-ICD for each case.

CORRELATION BETWEEN THE KINETIC ENERGIES OF THE RESONANT AUGER ELECTRONS AND THE ICD ELECTRONS

A weak, diagonally shaped feature due to RA-ICD can be observed in Fig. S3. It can straightforwardly be interpreted as a constant sum of resonant Auger electron and ICD electron. The slower the Auger electron, the more internal energy is still stored in the ion, and the more energy can be released in the transition to the ground state. Consequently, the maximum energy of the ICD electron increases. We emphasize this by dividing the coincidence filter condition, which has been used to obtain the spectra in Fig. 4 of the main manuscript, in two parts, the low- and high-energy parts of the spectator resonant Auger peak. Both corresponding RA-ICD spectra are shown in Fig. S4 for the $2p_{3/2} \rightarrow 3d$ resonance at 349.25 eV. As expected, a weak but significant shift of intensity in the RA-ICD feature according to the selected range of the Auger peak can be observed.

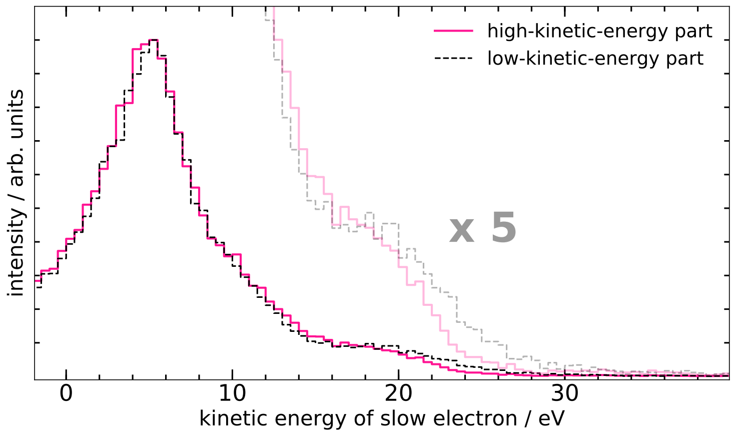

FIG. S4. RA-ICD electron spectra on the $2p_{3/2} \rightarrow 3d$ resonance at 349.25 eV for narrow coincidence conditions containing the low- or high-kinetic-energy part of the resonant Auger spectrum. A correlation between the kinetic energy of the Auger electrons and the RA-ICD electrons can be observed, namely, the lower the Auger electron kinetic energy, the higher the RA-ICD electron energy and *vice versa*. This correlation describes the release of a constant amount of energy in the two-step transition from the inner-shell vacancy to the final state.
